# *Bartonella quintana* Infection in Kidney Transplant Recipients from Donor Experiencing Homelessness, United States, 2022

**DOI:** 10.3201/eid3012.240310

**Published:** 2024-12

**Authors:** Amy M. Beeson, Shannan N. Rich, Michael E. Russo, Julu Bhatnagar, Rebecca N. Kumar, Jana M. Ritter, Pallavi Annambhotla, Moe R. Takeda, Kira F. Kuhn, Prishanya Pillai, Marlene DeLeon-Carnes, Rebecca Scobell, Maheswari Ekambaram, Rachel Finkel, Sarah Reagan-Steiner, Roosecelis B. Martines, Rohit S. Satoskar, Gayle M. Vranic, Raji Mohammed, Gloria E. Rivera, Kumarasen Cooper, Heba Abdelal, Marc Roger Couturier, Benjamin T. Bradley, Alison F. Hinckley, Jane E. Koehler, Paul S. Mead, Matthew J. Kuehnert, Joel Ackelsberg, Sridhar V. Basavaraju, Grace E. Marx

**Affiliations:** Centers for Disease Control and Prevention, Fort Collins, Colorado, USA (A.M. Beeson, S.N. Rich, A.F. Hinckley, P.S. Mead, G.E. Marx); The Children's Hospital of Philadelphia, Philadelphia, Pennsylvania, USA (M.E. Russo, M.R. Takeda, K.F. Kuhn, R. Scobell, M. Ekambaram, R. Finkel, H. Abdelal); Perelman School of Medicine at University of Pennsylvania, Philadelphia (M.E. Russo, M.R. Takeda, K. Cooper); Centers for Disease Control and Prevention, Atlanta, Georgia, USA (J. Bhatnagar, J.M. Ritter, P. Annambhotla, M. DeLeon-Carnes, R.S. Satoskar, M.J. Kuehnert, S.V. Basavaraju); MedStar Georgetown University Hospital, Washington, DC, USA (R.N. Kumar, P. Pillai, S. Reagan-Steiner, R.B. Martines, G.M. Vranic); Duke University Medical Center, Durham, NC, USA (G.M. Vranic); Lincoln Medical Center, New York, NY, USA (R. Mohammed); New York City Department of Health and Mental Hygiene, New York (G.E. Rivera, M.J. Kuehnert, J. Ackelsberg); University Hospitals Cleveland Medical Center, Cleveland, Ohio, USA (H. Abdelal); ARUP Laboratories, Salt Lake City, Utah, USA (M.R. Couturier, B.T. Bradley); University of Utah, Salt Lake City (M.R. Couturier, B.T. Bradley); University of California, San Francisco, California, USA (J.E. Koehler).

**Keywords:** *Bartonella quintana*, transplant, homelessness, bacteria, United States

## Abstract

*Bartonella quintana* infection can cause severe disease that includes clinical manifestations such as endocarditis, chronic bacteremia, and vasoproliferative lesions of the skin and viscera. *B. quintana* bacteria is transmitted by the human body louse (*Pediculus humanus corporis*) and is associated with homelessness and limited access to hygienic services. We report *B. quintana* infection in 2 kidney transplant recipients in the United States from an organ donor who was experiencing homelessness. One infection manifested atypically, and the other was minimally symptomatic; with rapid detection, both recipients received timely treatment and recovered. *B. quintana* was identified retrospectively in an archived donor hematoma specimen, confirming the transmission link. Information about the organ donor’s housing status was critical to this investigation. Evaluation for *B. quintana* infection should be considered for solid organ transplant recipients who receive organs from donors with a history of homelessness or of body lice infestation.

*Bartonella quintana* is a small, facultatively intracellular, gram-negative bacillus that is transmitted to humans through the feces of an infected human body louse (*Pediculus humanus corporis*) (*1*). *B. quintana* infection was first described among soldiers living in poor hygienic conditions during World War I and became known as trench fever (*2*). In the United States and Europe, *B. quintana* infection has become increasingly linked to urban homelessness, a consequence of crowded living conditions and inconsistent access to clean clothes, showers, and laundry facilities (*3*,*4*).

The clinical manifestations of *B. quintana* infection range from asymptomatic to life-threatening. The most frequently described manifestations are endocarditis, bacteremia, and bacillary angiomatosis, a vasoproliferative disorder that primarily affects the skin but can also affect bones, liver, and spleen (*5*,*6*). Prolonged *B. quintana* bacteremia may be asymptomatic or pauci-symptomatic over several years (*7*). *B. quintana* manifestations vary by immune status; bacillary angiomatosis occurs mostly in severely immunocompromised patients (*8*).

*B. quintana* infection can be identified by serologic testing, culture, or molecular methods (*9*). Current serologic tests do not reliably differentiate *B. quintana* from other *Bartonella* species, including the more common *B. henselae*, which causes cat-scratch fever. *B. quintana* is slow-growing, and culture is aided by prolonged incubation and specialized techniques (*10*–*12*). Molecular diagnostic tests for *Bartonella* are more sensitive than culture but are currently only available from a few commercial laboratories and vary in their ability to distinguish between *Bartonella* species (*13*).

*Bartonella* infection has previously been found in solid organ transplant recipients (*14*,*15*). In most of those cases, *B. henselae* was the suspected or confirmed causative pathogen, and it was presumed the recipient acquired the infection after exposure to household or feral cats (*14*,*15*). Very rarely, donor-derived *Bartonella* infection has been documented: a *B. henselae* infection (*16*), a renal transplant recipient in the Czech Republic with *B. quintana* infection (*17*), a suspected cluster of *Bartonella* infections in 3 recipients (heart, liver, and kidney) from a donor in the United States (*18*), and a recent cluster among 6 recipients from unhoused seropositive donors in Alberta, Canada (*19*).

In December 2022, a *Bartonella* infection was diagnosed in a kidney transplant recipient in the United States with no identifiable risk factors, spurring a complex investigation to determine the species and source of infection. Molecular testing eventually confirmed *B. quintana* in the recipient. Active screening identified *B. quintana* bacteremia in a second kidney transplant recipient from the same organ donor, and *B. quintana* was detected in an archived, formalin-fixed paraffin-embedded (FFPE) hematoma specimen from the organ donor. We describe the *B. quintana* infections in this organ donor who was experiencing homelessness and the 2 kidney recipients and discuss the diagnostic and treatment considerations among transplant recipients and public health implications. 

## Methods

### Ethics Considerations

This investigation was conducted as part of the Centers for Disease Control and Prevention (CDC) and the New York City (NYC) Department of Health and Mental Hygiene routine public health surveillance and disease investigation activities. The CDC Human Subjects Review committee reviewed this investigation and deemed it to be nonresearch and exempted from full review. Recipient A’s guardian and recipient B gave informed consent to include a summary of their cases in this report.

## Case Description for Recipient A

In late 2022, a 16-year-old male patient who had received a kidney transplant 3 months before was hospitalized because of progressive abdominal pain over the previous 2 weeks, loose stools, intermittent fevers, and 2 days of cough and posttussive emesis. The patient had chronic kidney disease secondary to atypical hemolytic uremic syndrome as a child and had received his first deceased donor kidney transplant >10 years before. After graft failure in 2019 because of antibody-mediated rejection, he received a second deceased donor transplant in 2022 with antithymocyte globulin induction. At the time of admission, 3 months after transplant, the patient’s immunosuppressive regimen included tacrolimus, mycophenolate, and prednisone. Since transplantation, he had been receiving prophylactic trimethoprim/sulfamethoxazole and valganciclovir.

**Figure 1:**
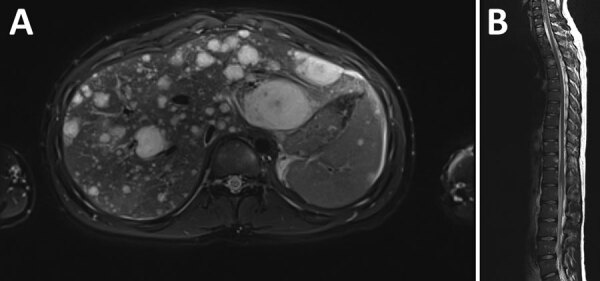
Imaging of a kidney transplant recipient with a *Bartonella quintana* infection linked to a donor who experienced homelessness, United States, 2022. A) Multiple enhancing T2 hyperintense lesions throughout the hepatic parenchyma. B) Numerous enhancing T2 hyperintense lesions affecting cervical, thoracic, and lumbar vertebrae.

Recipient A was febrile and tachycardic on initial examination. We noted right upper quadrant and epigastric tenderness and mild abdominal distension; no hepatosplenomegaly, lymphadenopathy, or skin lesions were observed. Laboratory testing showed creatinine mildly elevated from baseline (from 0.8 to 1.2 mg/dL), low hemoglobin (10.2 g/dL, reference range 13.0–16.0 g/dL), neutropenia (absolute neutrophil count, 950 cells/µL, reference range 1,540–7040 cells/µL), lymphopenia (absolute lymphocyte count, 520 cells/µL, reference range 970–3260 cells/µL), elevated C-reactive protein (5.2 mg/dL, reference range 0.0–0.9 mg/dL), and unremarkable alanine transaminase (19 U/L) and aspartate transaminase (36 U/L) values. His abdominal pain progressively worsened. Signs of peritonitis developed 4 days after admission, and we found an intraperitoneal hemorrhage that required resection. Magnetic resonance imaging revealed multiple T2 hyperintense enhancing lesions in his liver, spleen, and every vertebral body (Figure 1). A ruptured hepatic lesion was believed to be the source of the hemorrhage.

**Figure 2:**
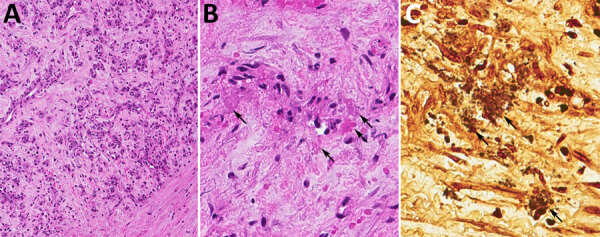
Liver biopsy of a kidney transplant recipient with a *Bartonella quintana* infection linked to a donor who experienced homelessness, United States, 2022. A) Fibrovascular proliferation accompanied by few inflammatory cells. Magnification ×200. B) Myxoid stroma that contains clumps of granular material (arrows). Magnification ×400. C) Warthin-Starry stain highlights clumps of bacilli (arrows). Magnification ×400.

We initiated empiric antimicrobial treatment at admission with cefepime and vancomycin; the patient received multiple β-lactam medications during the first week. An extensive evaluation for infectious etiologies was unremarkable, which included *B. henselae* serologic testing that was negative at admission. Transthoracic echocardiogram revealed no abnormalities. We suspected posttransplant lymphoproliferative disease, but a needle biopsy of recipient A’s liver was nondiagnostic. We performed an excisional biopsy of 1 liver lesion (Figure 2) that showed histopathologic findings of a vasoproliferative lesion consistent with bacillary angiomatosis, including clusters of bacteria seen on Warthin-Starry stain, prompting targeted testing for *Bartonella*. Results of *Bartonella* PCR conducted by ARUP Laboratories (Salt Lake City, UT, USA) were positive 30 days after admission (Table 2), and we initiated *Bartonella*–directed therapy. The laboratory-developed test used by ARUP Laboratories is a real-time PCR to detect *Bartonella* species from the liver and blood. The assay uses primers and probes specific for the heat shock protein gene of *Bartonella* species. The assay is validated to detect both *B. henselae* and *B. quintana* with a limit of detection of 643 copies/mL. After nucleic acid extraction, specimens were amplified on the QuantStudio 12k Flex (Thermo Fisher Scientific, https://www.thermofisher.com) instrument using the GoTaq Probe qPCR Master Mix (Promega, https://www.promega.com) with primers and probes manufactured by ELITech (https://www.elitechgroup.com). Specimens were considered positive if cycle threshold was <36.

**Table 2:** Test results other than serology from kidney transplant recipients and donor with *Bartonella quintana* infection, United States, 2022*

Source	Time posttransplant specimen collection, wk	Specimen type	Test performed (performing laboratory)	Result
Recipient A	16	Liver tissue (FFPE)	*Bartonella* spp. PCR (ARUP Laboratories)	Positive
	16	Liver tissue (FFPE)	*Bartonella* spp. PCR (CDC)	Positive
	16	Liver tissue (FFPE)	*Bartonella *spp*.* sequencing (CDC)	*B. quintana*
	16	Liver tissue	Fungal, aerobic, and anaerobic tissue culture	No growth
	19	Serum	*Bartonella* spp. PCR (ARUP Laboratories)	Positive
	19	Whole blood	Blood culture	No growth after 5 d
Recipient B	26	Whole blood	*Bartonella* spp. PCR (ARUP Laboratories)	Positive
	54	Serum	*Bartonella* spp. PCR (ARUP Laboratories)	Negative
Donor	Prior to transplant	Whole blood	Blood culture	No growth after 5 d
	Archived specimen	Serum	*Bartonella* spp. PCR (ARUP Laboratories)	Negative
	Archived specimen	Hematoma (FFPE)	*Bartonella* spp. PCR (CDC)	Positive
	Archived specimen	Hematoma (FFPE)	*Bartonella* spp. sequencing (CDC)	*B. quintana*

*CDC, Centers for Disease Control and Prevention; FFPE, formalin-fixed paraffin-embedded.

Recipient A did not have any known exposures that would confer risk of either *B. henselae* or *B. quintana* infection, such as contact with felines or persons experiencing homelessness or a history of body lice infestation. We filed a report with the Organ Procurement and Transplantation Network for investigation by the ad hoc Disease Transmission Advisory Committee. In addition, we submitted a FFPE liver tissue specimen to CDC for further histopathologic evaluation and laboratory testing (Table 2). Histopathologic features were compatible with bacillary angiomatosis. Results of an immunohistochemical assay for *B. henselae* were negative, but results of a conventional *Bartonella* species-specific PCR on DNA extracted from liver tissue were positive, and Sanger sequencing of the PCR products identified *B. quintana*. We conducted a conventional *Bartonella* species-specific heminested PCR targeting the riboflavin synthase-encoding gene on DNA extracted from FFPE tissue (*20*). We confirmed positive results by using sequencing.

The patient’s fever and abdominal pain resolved within 1 week of starting *Bartonella*-specific antimicrobial drugs. Results of an eye examination including slit lamp and dilated funduscopic examination were unremarkable. The patient experienced gastrointestinal side effects from the first-line treatment doxycycline, prompting a switch to azithromycin. He then experienced a slight increase in mild preexisting tinnitus that did not progress during the remainder of treatment. He was treated for a total of 23 weeks. Repeat imaging showed nearly complete resolution of liver, spleen, and vertebral lesions with no residual enhancing lesions. *B. henselae* serologic testing was repeated twice, becoming positive on the third test with an IgG titer of 1:512 (Table 1). *B. quintana* serologic testing was not readily available, but the *B. henselae* assay that was used is known to cross-react with *B. quintana*. The patient remained without symptoms during an 8-month follow-up.

**Table 1:** Serologic test results from kidney transplant recipients and donor with *Bartonella quintana* infection, United States, 2022*

Source	Posttransplant specimen collection, weeks	Test performed	Result
Recipient A	14	*B. henselae* IgG	Negative
		*B. henselae* IgM	Negative
	19	*B. henselae* IgG	Negative
		*B. henselae* IgM	Negative
	35	*B. henselae* IgG	1:512
		*B. henselae* IgM	Negative
Recipient B	26	*B. henselae* IgG	1:16,384
		*B. henselae* IgM	Negative
	26	*B. quintana* IgG	1:2,048
		*B. quintana* IgM	1:64
	30	*B. henselae* IgG	1:32,768
		*B. henselae* IgM	Negative
	30	*B. quintana* IgG	1:4,096
		*B. quintana* IgM	1:128
	44	*B. henselae* IgG	1:1,024
		*B. henselae* IgM	Negative
	44	*B. quintana* IgG	1:512
		*B. quintana* IgM	Negative
	54	*B. henselae* IgG	1:1,024
		*B. henselae* IgM	Negative
	54	*B. quintana* IgG	1:256
		*B. quintana* IgM	Negative
	63†	*B. henselae* IgG	1:64
		*B. henselae* IgM	Negative
	63†	*B. quintana* IgG	Negative
		*B. quintana* IgM	Negative
Donor	Archived specimen	*B. henselae* IgG	1:256
		*B. henselae* IgM	Negative
	Archived specimen	*B. quintana* IgG	Negative
		*B quintana* IgM	Negative

*All serologic testing was performed at ARUP Laboratories (Salt Lake City, Utah, USA). †Recipient B’s 63-week samples were not sent to ARUP Laboratories. Reference ranges for *B. henselae* and *B quintana* titers: IgG <1:64, negative; 1:64–1:128, equivocal; >1:256, positive; IgM <1:16, negative; >1:16, positive.

## Investigation

Shortly after recipient A began antimicrobial treatment for a *Bartonella* infection, CDC learned of the case through the Emerging Infections Network (*21*) and initiated a collaborative investigation including clinicians and public health authorities in several states, the organ procurement organization, and laboratory partners. The investigation revealed that 2 organs (left and right kidneys) had been recovered from a common donor and transplanted into 2 organ recipients. In addition, bone was recovered from the donor for processing as allograft tissue.

CDC contacted the transplant team caring for the second kidney recipient (recipient B), and the patient was promptly evaluated. In addition, CDC and the NYC Department of Health and Mental Hygiene collaborated to evaluate the possibility of donor-derived *Bartonella* infection through a review of the donor’s medical record, phone outreach to the donor’s listed contacts, and testing of residual specimens.

## Case Description for Recipient B

In mid-2022, a 54-year-old male patient underwent a third renal transplant for advanced nephropathy secondary to type 2 diabetes mellitus. Recipient B was evaluated 5 months after transplantation in an infectious disease clinic after the transplant team learned of the infection in recipient A. Recipient B reported feeling well other than bilateral hand and knee arthralgias that had begun shortly after the transplant. His immunosuppressive regimen consisted of tacrolimus, mycophenolate, and prednisone. After the transplant, he received posttransplant prophylaxis with trimethoprim/sulfamethoxazole for 3 months. Recipient B reported 1 pet dog and 3 cats. He reported no history of experiencing homelessness, incarceration, body lice infestation, or contact with persons experiencing homelessness.

Recipient B’s physical examination was unremarkable and showed no audible cardiac murmur. We noted hyperpigmented macules on bilateral palms. Laboratory testing showed no acute changes in renal or hepatic function tests, blood chemistry, complete blood count, or C-reactive protein. Serum *Bartonella* PCR results were positive, and the patient was found to have elevated antibody titers (*B. henselae* IgG 1:16,384; *B. henselae* IgM <1:16; *B. quintana* IgG 1:2,048; *B. quintana* IgM 1:64). *B*. *henselae* and *B*. *quintana* IgG were detected at ARUP Laboratories by using the BIOCHIP Mosaic (EuroImmun, https://www.euroimmun.com) indirect immunofluorescence antibody assay (Table 2). Serum specimens were diluted to 1:64 for screening. We serially diluted positive specimens to endpoint reactivity to determine a final titer. Specimens with titers <1:64 were considered negative. Titers of 1:64–1:128 were considered equivocal and required subsequent convalescent testing for confirmation. Titers >1:128 were considered positive. We detected *B*. *henselae* and *B*. *quintana* IgM by using indirect immunofluorescence antibody assay (DiaSorin, https://int.diasorin.com). We diluted serum specimens to 1:16 and further titrated specimens with reactivity at 1:16 to endpoint reactivity. We reported titers <1:16 as negative and titers >1:16 as positive. Computed tomography scans without contrast of the abdomen and pelvis showed an edematous, ill-defined transplanted kidney without other acute abnormalities. Dilated eye examination did not reveal retinal abnormalities. Transthoracic and transesophageal echocardiography showed mild thickening of the mitral valve leaflets and moderate thickening of aortic valve leaflets, without definite vegetations. The aortic valve thickening was more prominent compared with a study completed 2 years prior. Recipient B was referred to dermatology for evaluation of palmar lesions, which were not believed to be consistent with Janeway lesions.

Although recipient B did not meet the modified Duke criteria for definite endocarditis, on the basis of the valvular changes, positive *Bartonella* PCR in blood, and potential exposure through transplantation, we initiated treatment for possible *Bartonella* endocarditis with oral doxycycline (100 mg 2×/d) and oral azithromycin (500 mg 1×/d initial dose, then 250 mg 1×/d) (*22*). Shortly after treatment initiation, the patient’s joint pain resolved. After 3 weeks, we stopped the azithromycin. We added rifampin for 6 weeks, during weeks 11–17 of treatment, and then discontinued. Because of the risk for subtherapeutic levels of tacrolimus with coadministration of rifampin, we tapered and then discontinued the tacrolimus and replaced it with belatacept.

Erythrocyte sedimentation rate and C-reactive protein were unremarkable and repeat antibody titers showed increased *B. henselae* and *B. quintana* titers 1 month after treatment initiation. Approximately 4 months after treatment initiation, the titer values had decreased (Table 1). Recipient B has remained on oral doxycycline (100 mg 2×/d) for maintenance to date. Repeat echocardiography is planned.

## Donor Description

Medical records from the organ donor’s terminal hospitalization documented a traumatic brain injury that led to brain death despite neurosurgical interventions. A history of alcohol use disorder was noted; the donor had no other known immunocompromising conditions. No concerns for preexisting infection were noted during the terminal hospitalization. The donor underwent 2 surgical evacuations of a subdural hematoma that required multiple blood transfusions. Postoperatively, the donor experienced tachycardia, fever with a temperature >106°F, and leukocytosis. Sympathetic nervous system hyperactivity was a suspected cause of the marked hyperthermia. Despite aggressive treatment, the patient was determined to have very limited chance of recovery. The next of kin decided to transition to comfort-focused care, and the patient died shortly afterward.

The donor’s hospital record and organ donation records did not explicitly note a history of homelessness or body lice infestation; however, after the recipients became ill, independent interviews with 2 people who knew the donor revealed a history of unsheltered homelessness in the months before the terminal hospitalization. Records at NYC’s Department of Homeless Services did not indicate that the donor had ever been registered for services provided by the agency.

We tested a frozen, archived serum sample from the deceased donor for *Bartonella* by using PCR, which was negative. We also tested the donor’s serum for antibodies to *B. henselae* and *B. quintana*, which was positive only for *B. henselae* IgG with a titer of 1:256. We located a subdural hematoma specimen from the donor that had been collected during a neurosurgical procedure and preserved. The hematoma showed erythrocytes admixed with fibrin, abundant neutrophils, and cellular debris. We conducted a *Bartonella*-specific PCR on DNA extracts from this FFPE specimen, and results were positive (Table 2). We conducted Sanger sequencing of the PCR products and identified *B. quintana *(Table 2).

The bone that had been recovered from the donor and processed as allograft tissue was quarantined when illness was first identified in recipient A. The bone specimens were not tested because there are few validated tests for this specimen type; the specimens were destroyed at the conclusion of this investigation.

## Discussion

Our findings suggest *B*. *quintana* transmission through solid organ transplantation in 2 recipients from 1 donor, with species-specific confirmation of infection in the donor and recipient A. We suspect the organ donor in this case acquired *B. quintana* from a body lice infestation while experiencing homelessness. In recent decades, numerous cases and clusters of *B. quintana* infection have been described among persons experiencing homelessness in urban areas (*4*,*23*–*29*).

The organ donor in this cluster had slept outdoors in the months before the terminal hospitalization and had a history of alcohol use disorder. Among persons experiencing homelessness, those without consistent access to shower and laundry facilities or clean clothes are at risk for body lice infestation and *B. quintana* systemic infection. In the United States, the prevalence of unsheltered homelessness is increasing, and unsheltered homelessness has been linked to body lice infestation and *B. quintana* in San Francisco and NYC (*29*,*30*). Alcohol use disorder has also frequently been associated with *B. quintana* infection (*4*,*27*).

The cases in this study came to prompt attention because both recipients were engaged with posttransplant care teams and received a high level of care. However, most patients who have *B. quintana* infections do not receive the same level of attention or prompt treatment. This cluster serves as a reminder that this emerging infection still affects many on the margins of society and that public health measures are needed, including improved hygienic services, healthcare access, substance use treatment programs, and interventions to address the root causes of homelessness.

Limited clinician awareness of the risk factors for *B. quintana* transmission, its variable clinical manifestations, and laboratory diagnostic challenges contribute to the likely underdiagnosis of *B. quintana* infection. Those and other factors might also cause delayed or missed identifications in organ transplant recipients, a population with more consistent healthcare access but at risk for atypical disease manifestations and severe illness or death from *Bartonella* infections because of immunosuppression. Of note, both recipients in this cluster displayed unusual clinical manifestations of *B. quintana*. Recipient A’s case demonstrates the potential for atypical and severe manifestations of *B. quintana* during immunosuppression. Hepatosplenic manifestations of *B. quintana* have rarely been described and are much more characteristic of *B. henselae* infection (*6*); the co-occurrence of osteomyelitis with bacillary angiomatosis of the liver is highly unusual. Recipient B’s case demonstrates the potential for indolent, pauci-symptomatic *B. quintana* infection and chronic bacteremia among transplant recipients. Recipient B’s infection and probable endocarditis was only found through active screening performed as part of this public health investigation, because he did not report fevers or have symptoms of endocarditis that would typically prompt echocardiography. Of note, *Bartonella* infection occurred despite prolonged use of prophylactic trimethoprim/sulfamethoxazole in both recipients; trimethoprim/sulfamethoxazole is sometimes recommended as treatment for *B. henselae* infection, but treatment failure has been documented in patients with bacillary angiomatosis (*8*). Clinicians caring for transplant recipients should consider *B. quintana* infection in organ transplant recipients with clinical manifestations including prolonged fever, culture-negative endocarditis, or bacillary angiomatosis involving the skin, liver, spleen, or bone.

The cases in this study also underline the diagnostic challenges specific to solid organ transplant recipients. Serologic testing, a mainstay of *Bartonella* diagnosis, may remain negative for a prolonged interval because of immunosuppression. Blood product transfusion during or after transplantation can cloud the interpretation of test results because dilution can cause falsely negative results, whereas immunoglobulins acquired from donor plasma can result in false positive results (*31*,*32*). Serologic tests and many molecular tests for *Bartonella* infections lack species specificity. Identification of specific *Bartonella* species has implications for prevention because those species have distinct transmission pathways and epidemiologic risk factors. In the United States, *B. henselae* is vectored by the cat flea, whereas *B. quintana* is transmitted by the human body louse. Infections with other, less common *Bartonella* species have been associated with other vectors and animals. Because of the unreliability of serologies and the difficulty of isolating *B. quintana* in culture, testing by using molecular diagnostic methods is particularly necessary if *B. quintana* is suspected in solid organ transplant recipients; providers should note that sensitivity may vary according to the specific assay and sample type.

For investigations of suspected transplant-associated transmission, obtaining FFPE tissue specimens when available from the recipient(s) and donor have frequently been used to help establish tissue-based diagnoses that can help guide patient management and inform epidemiologic investigations (*33*–*35*). Evaluation of such tissue specimens can be especially useful when conventional specimens such as serum and blood are unavailable or when results of testing those specimens are negative or not fully interpretable.

Because laboratory testing for *B. quintana* can be resource-intensive and because of the limitations of existing tests, universal screening of organ and tissue donor specimens for *B. quintana* or *Bartonella* infection before transplantation is neither feasible nor advisable. *B. quintana* is treatable, and positive test results in a prospective donor should not preclude transplantation of a lifesaving organ. As an alternative to universal donor screening, data on the housing status of organ donors (such as a history of homelessness or current homelessness) collected from the donor’s next of kin might prove useful to clinicians managing potential donor-derived infections in transplant recipients; unfortunately, those data are not currently collected systematically (*36*). When a history of homelessness or body louse infestation is recognized in a donor, testing donor samples with blood culture, serology, molecular assays, or a combination of those methods should be considered. For recipients from organ donors who test positive, periodic testing, echocardiography, or even preemptive treatment might be relevant to prevent late complications of *B. quintana* infection, even if recipients are asymptomatic.

Although treatment of both organ transplant recipients in this cluster has been successful to date, management of *B. quintana* infection in the setting of transplantation has distinct challenges. Effective antimicrobial drug durations for immunocompromised persons are not clearly established, although the experience with treatment of *Bartonella* infection in severely immunocompromised persons living with HIV has demonstrated that lengthy antimicrobial drug treatment is necessary to prevent relapse. Antimicrobial drugs effective against *B. quintana* include doxycycline, rifampin, aminoglycosides, and macrolides (*37*). After kidney transplantation, rifampin use can be challenging because of interactions with common immunosuppressive medications used to prevent transplant rejection, and aminoglycoside use may be limited by nephrotoxicity.

The first limitation of this report is that, although the NYC Department of Health and Mental Hygiene did not find records to indicate that the donor sought care for symptoms suggestive of bartonellosis before death, the donor could have sought care at a facility not captured by the regional health information exchange organizations that were queried. Therefore, it remains unknown whether the donor experienced signs or symptoms of *B. quintana* infection before death and whether there might have been missed opportunities for earlier diagnosis. Second, although the donor had epidemiologic risk factors for the acquisition of *B. quintana* through infected body lice, it is also possible that the donor acquired the infection through blood transfusion (*18*) because transmission through blood transfusion has been demonstrated (*38*). Finally, *Bartonella* species confirmation for recipient B and molecular sequencing of donor and recipient specimens could have provided further evidence of donor-to-recipient transmission. We attempted additional 16S sequencing testing on the liver tissue sample from recipient A and the PCR-positive blood sample from recipient B, but results were indeterminate.

 In conclusion, this investigation of a donor-derived cluster of *B. quintana* infections in 2 solid organ transplant recipients yields lessons for public health practitioners and clinicians. *B. quintana* is a growing public health problem in the United States, but it is not nationally notifiable and is underrecognized. In this investigation, information sharing and collaboration among clinicians, laboratorians, and public health authorities led to timely testing, treatment, and clinical improvement for both organ recipients. Improving diagnosis, treatment, and prevention of *B. quintana* infections in the United States depends on heightened awareness of this enigmatic pathogen, including its potential to cause illness in solid organ transplant recipients.
